# De-identified data quality assessment approaches by data vendors who license data to healthcare and life sciences researchers

**DOI:** 10.1093/jamiaopen/ooac093

**Published:** 2022-11-02

**Authors:** C Erwin Johnson, Daniel Colquhoun, Daniel A Ruppar, Sascha Vetter

**Affiliations:** Policy Evidence Research (Global Market Access), Merck & Co., Inc., Kenilworth, New Jersey, USA; Customer Research, Frost & Sullivan, Toronto, Ontario, Canada; Health & Life Sciences, Frost & Sullivan, San Antonio, Texas, USA; Customer Research, Frost & Sullivan, San Antonio, Texas, USA

**Keywords:** qualitative research, data quality assessment, real-world data (RWD), real-world evidence (RWE), interoperability, de-identified data, anonymized data, data quality assessment standards, electronic health records

## Abstract

**Objective:**

To gain insights into how data vendor companies (DVs), an important source of de-identified/anonymized licensed patient-related data (D/ALD) used in clinical informatics research in life sciences and the pharmaceutical industry, characterize, conduct, and communicate data quality assessments to researcher purchasers of D/ALD.

**Materials and Methods:**

A qualitative study with interviews of DVs executives and decision-makers in data quality assessments (*n* = 12) and content analysis of interviews transcripts.

**Results:**

Data quality, from the perspective of DVs, is characterized by how it is defined, validated, and processed. DVs identify data quality as the main contributor to successful collaborations with life sciences/pharmaceutical research partners. Data quality feedback from clients provides the basis for DVs reviews and inspections of quality processes. DVs value customer interactions, view collaboration, shared common goals, mutual expertise, and communication related to data quality as success factors.

**Conclusion:**

Data quality evaluation practices are important. However, no uniform DVs industry standards for data quality assessment were identified. DVs describe their orientation to data quality evaluation as a direct result of not only the complex nature of data sources, but also of techniques, processes, and approaches used to construct data sets. Because real-world data (RWD), eg, patient data from electronic medical records, is used for real-world evidence (RWE) generation, the use of D/ALD will expand and require refinement. The focus on (and rigor in) data quality assessment (particularly in research necessary to make regulatory decisions) will require more structure, standards, and collaboration between DVs, life sciences/pharmaceutical, informaticists, and RWD/RWE policy-making stakeholders.

## INTRODUCTION

Real-world data (RWD) is the currency of transformation in healthcare research. RWD used to create real-world evidence (RWE) is growing across the healthcare research ecosystem from drug development to policymaking.^1–7^ Researchers in life sciences/pharma purchase de-identified/anonymized licensed patient-related data (D/ALD) mainly to conduct health economic and outcomes research for their pharmaceutical products. For the purposes of this discovery study, de-identified/anonymized data refer to patient data sold/licensed by data vendor companies (DVs) as a business product and services offering from which all identifying information has been removed so that the individual data or information of a customer cannot be associated with that customer without extraordinary effort.^8^

RWD quality is a priority for research-funding institutions, eg, the National Institutes of Health.^9^ Data quality assessment frameworks exist and are applied to large data sets used to conduct research.^10^^,^^11^ In addition, collaborative partnerships have emerged to provide guidance to address data quality for RWD.^12^ It is not known how DVs use these frameworks to assess the quality of their D/ALD. The importance of data quality assessment was recently heightened as part of a publication retraction in *The Lancet* in a study related to hydroxychloroquine use for the treatment of COVID-19.^13^ The journal’s editorial board could not assess the DVs data completeness (a data quality assessment dimension) assessment process. Data transparency (another data quality assessment dimension) limitations also contributed to the retraction. This example highlights the need to assess the quality of D/ALD, or any RWD, purchased by life sciences/pharma researchers who conduct research to generate RWE. No peer-reviewed studies could be identified to understand how DVs assess the quality of D/ALD they sell/license to life sciences/pharma researchers, or how the quality of D/ALD should be regulated. Therefore, we conducted a discovery study to understand how data quality is defined, assessed, and communicated, along with the use of interoperability resources/technologies for D/ALD licensed to life sciences/pharma researchers from the DVs perspective.

## METHODS

### Design

As a discovery investigation, a qualitative study design was used.^14–17^ We identified a purposive sample frame (a non-probability sample) of 38 DVs through the following organic approaches:

- Proprietary and syndicated gray literature research that identified, via a mix of desk-based secondary and primary interviews with vendors in industry, key vendors in the healthcare database vendor industry that provide D/ALD.^18^
- Targeted Google^TM^ search engine web searches using keywords and applicable acronyms: data vendor(s), anonymized licensed data, RWE, RWD, known data.
- Identified data vendor company names were searched for links to other DVs. The names of the DVs used in search terms are not included in this publication to protect the confidentiality of the study participants. Anonymity was promised. This allowed participants to share honestly without concern that shared information would disadvantage their company.

### Respondent screening and profile

**Figure 1.**
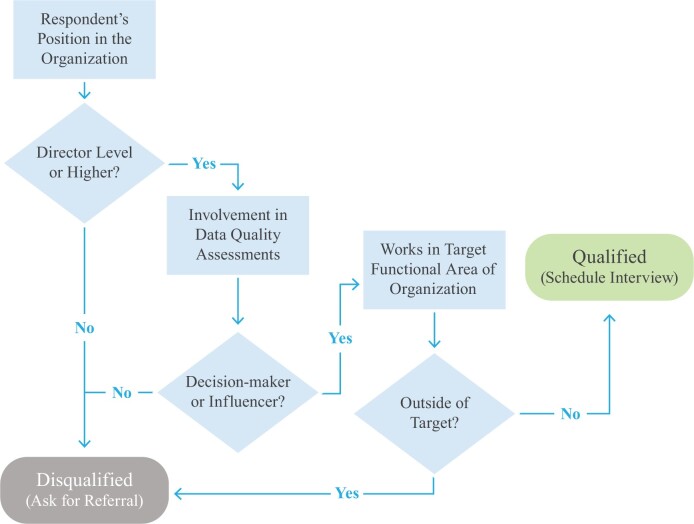
Study participant screening.

Each unpaid volunteer participant completed a screening questionnaire and was evaluated by a set process (Figure 1) inclusive of: title of director level or higher, involvement in data quality assessments as a decision-maker or influencer, and works in a target functional area of the organization (eg, data management, data science, legal, research, and engineering). This study did not seek individual or population-level data and is not human subjects research. Therefore, Institutional Review Board approval was not sought or obtained.

Contact information was developed using sources from purchased lists or desk-based research. A cold call was made to the organization, and a potential respondent with experience and background with data quality assessment was requested. Snowball sampling was used to strengthen the search for the most qualified respondent with each respondent.^19^ No additional DVs were identified via this additional technique outside of the original sample.

### Characteristics summary of respondents

Of the 12 study participants, 9 (75%) were director level and 3 (25%) were in an executive management or C-level position; 6 (50%) stated involvement (influence) in the decision-making related to their organization’s data quality assessments for D/ALD, but were not considered a key decision-maker and 2 (17%) stated that they were key decision-makers; 8 (67%) worked in either data sciences or research roles and 4 (33%) in data management or engineering roles (Table 1). No respondent was employed in a legal department.

**Table 1.** Characteristic summary of respondents (*n* = 12)

Characteristic	Category	Number	Percentage
Position	C-level executive	1	8
	Executive management such as vice president	2	17
	Director	9	75
Title	Chief scientific officer	1	8
	Vice president/senior vice president (any department)	2	17
	Director/associate director/senior director (any department)	8	67
	Senior principal (any department)	1	8
Involvement	I am one of the decision-makers for these assessments	2	17
	I influence the decision-making	6	50
	I do not take part in the decision-making but work with these types of assessments	4	33
Area of work	Data sciences	4	33
	Research	4	33
	Data management	3	25
	Engineering	1	8

We concluded that study participants represent a sample of the known DVs population in the United States. Our direct experience conducting research on the data vendor industry (eg, developing syndicated gray literature^18^^,^^20^^,^^21^) related to healthcare data usage for RWE facilitated access to, and garnered trust from, key leaders in approximately one-third of the prospective data vendor universe. A range of company sizes and personnel types were selected for interviews across tiers.

### Discussion guide development

Discussion guide questions were designed to capture important concepts related to the purchase of D/ALD from vendors to parties that use these data, based on the study team’s expertise related to RWD.

Interview questions were grouped as follows:

- Internal data quality assessment profile—how DVs define data quality and conduct internal data quality assessments of D/ALD sold to researchers;
- Interoperability profile—how DVs align their D/ALD assets to interoperability standards;
- Perceptions of life sciences/pharma partners—how DVs perceive interactions and partnerships with life sciences/pharma researchers that use D/ALD to conduct RWE research;
- RWE researcher engagement profile—how data vendor resources, strategies, and processes used to engage RWE researchers facilitate success, such as peer-reviewed manuscript publications, regulatory policy use, or other examples that demonstrate that RWE generated from high-quality RWD is trusted.

### Data collection

Twelve interviews, each with a unique company (Table 2), were conducted between November, 2020 and January, 2021. Each respondent was interviewed for 90 min (DC, SV) using a structured discussion guide. The sponsor of the study was not revealed to the respondent. The audio recording feature of the software application Zoom^®^ recorded each interview. Frost & Sullivan staff members programmed the questionnaire into the Voxco^®^^22^ computer-aided telephone interviewing software platform. This standard method of data collection was used to control the flow and execution of the interview and capture, via input from the interviewer, respondent answers.^23^

**Table 2.** Profile of participating data vendor companies (DVs)

DVs ID	Ownership status	Primary industry sector	Number of company employees Total	Revenue (USD)
1	Public	Business Products and Services	150 000+	1–50 Bn
2	Private	Healthcare	5000–15 000	500 Mn–1 Bn
3	Public	Healthcare	50 000–150 000	1–50 Bn
4	Private	Healthcare	<500	<100 Mn
5	Private	Healthcare	<500	<100 Mn
6	Subsidiary	Healthcare	500–5000	100–500 Mn
7	Public	Financial Services	15 000–50 000	50 Bn+
8	Subsidiary	Healthcare	150 000+	50 Bn+
9	Private	Healthcare	500–5000	<100 Mn
10	Public	Healthcare	15 000–50 000	1–50 Bn
11	Private	Healthcare	500–5000	100–500 Mn
12	Subsidiary	Business Products and Services	15 000–50 000	50Bn+

*Note*: Bn: Billion; Mn: Million; USD: United States Dollars.

### Data analysis

Interview recordings were transcribed by the same researchers who conducted the interviews (DC, SV). A qualitative thematic content analysis was conducted to analyze and categorize the interview data.^24^^,^^25^ First, the interview transcripts were manually coded.^26^ Verbatim print-outs of the transcripts were coded by systematically entering codes in the document margin by a researcher (DC) and the codes entered into a SPSS^®^ data repository. Second, a researcher (DC) conducted the initial analysis by utilizing tabulations of the codes to identify patterns^27^ or themes using WinCross^®^ tabulation software.^28^ Third, a second senior researcher (DAR) reviewed the results of the tabulation analysis, while conducting a separate review of the transcripts to ensure consensus on findings and completeness. This iterative process resulted in a finalized set of codes that both researchers agreed captured respondent answers. We report only the results from the data quality assessment and interoperability questions. The Supplementary Table provides illustrative quotes to support content and thematic findings.

## RESULTS

Results are grouped within 2 major themes, “Conducting data quality assessments on D/ALD” for which there are 4 subthemes, and “Addressing interoperability”, for which there are 2 subthemes.

### Conducting data quality assessments on D/ALD

#### Definition of data quality

For DVs, data quality is multi-dimensional (Supplementary Table, Quote A1) and includes: (1) the use of patient-level data from disparate sources, (2) processes that integrate patient data, (3) the importance of data structures, and (4) data cleanliness. Data quality was defined as “*the variance*” between actuality and intent-related concepts such as data produced, processes, and interpretation related to utility and outcomes (Supplementary Table, Quote A2). Most participants pointed to data quality as an organizational priority and have metrics in place for monitoring (Supplementary Table, Quote A3). Data quality metrics are mostly customized by the vendor and often for a particular customer or use case (Supplementary Table, Quote A4).

#### Sources of data

All participants sell electronic medical record (EMR) data to life sciences/pharma partners. More than two-thirds use patient-reported outcomes and claims data. About half use patient-generated health data of some type as well as data from Medicare Advantage plans (Table 3).

**Table 3.** Major sources of de-identified/anonymized licensed data (D/ALD) sold to pharma researchers

Data source	Percentage of respondents companies selling
Electronic medical records	100
Patient-reported outcomes	83
Claims	67
Patient-generated health data	58
Medicare advantage plans	58
Social determinants of health	42
Medicaid managed care organization plans	42
Immunization registry	33
Affordable Care Act plans	33
Wearable data	8

*Note*: All participants (*n* = 12). Multiple-mention response.

#### Approach and validation of data quality

Data quality assessment practices between DVs and research partners are iterative. They are (by stated necessity) as transparent as possible (Supplementary Table, Quote A5) within the boundaries of the DVs’ proprietary technology (Supplementary Table, Quote A6). The rigor and clarity (Supplementary Table, Quote A7) of these interactions can be important in terms of successful work outcomes. Almost half of the participants point to data quality as the main contributor to successful client collaborations.

**Figure 2.**
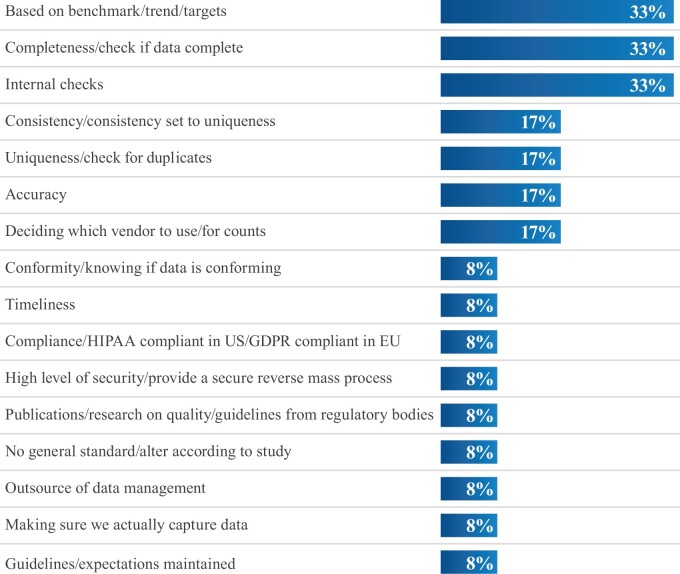
Data vendor data quality assessment guides or standards.

Key questions that research partners ask about D/ALD products include: (1) sample size, (2) type of data available for each patient, (3) data age, (4) missing data (Supplementary Table, Quote A8), (5) data governance (Supplementary Table, Quote A9), and (6) data structure. Specific questions about data quality during the purchase and evaluation period of D/ALD center on methodologies and systems, including those that are proprietary (Supplementary Table, Quote A10). Several participants reported that data accuracy and the longitudinal nature of the data (including from patients moving location, etc.) (Supplementary Table, Quote A11) were the most prevalent questions from pharma purchasers. All respondents use quantifiable measures or metrics to validate data quality, two-thirds developed in-house, which include reference to benchmarks, checks on completeness of data, identifying duplications, assessing data consistency, and timeliness (Figure 2) (Supplementary Table, Quote A12).

Human and machine-driven analytics are deployed throughout the process of data quality assessments. Even with advanced technology (eg, machine learning), DVs report that detailed and methodical inspection of data (often with manual intervention) is still a necessary part of the process (Supplementary Table, Quote A13).

Assessment processes typically begin with defining fields (Supplementary Table, Quote A14) and the use of statistical approaches to determine outliers, the extent of missing data, and their impact on data completeness. Character recognition and natural language processing (NLP) are examples of automation tools used to ensure data quality. Data first obtained by DVs is subject to a stepwise process to monitor integrity. Although there is variation in onboarding data, especially EMR data, some DVs take methodological and lengthy approaches (Supplementary Table, Quote A15) that leverage internal processes. Some DVs have limited means to assess data quality at a patient level and therefore trust the EMR data provider to provide quality assurance. All study participants recognize that EMR data are potentially flawed (Supplementary Table, Quote A16).

**Figure 3.**
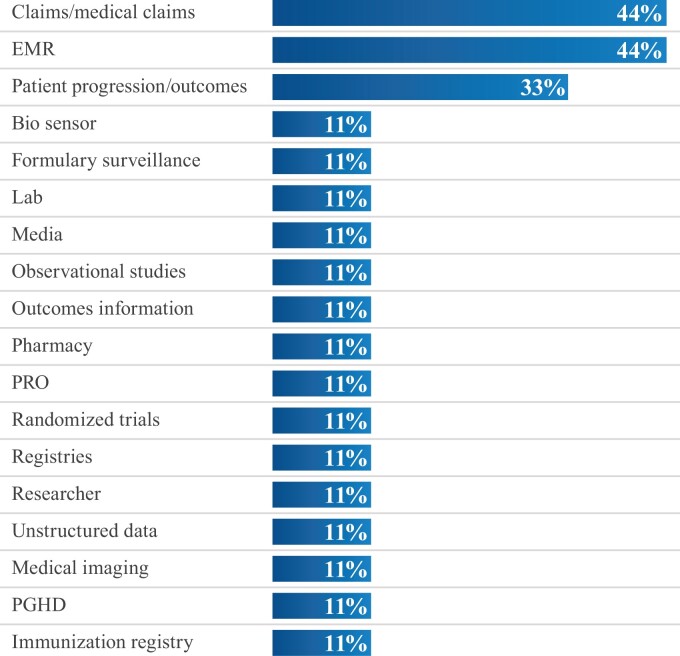
Data types difficult to evaluate for data quality.

EMR and claims data are the 2 types consistently stated as most difficult to validate for quality, followed by patient progression/outcomes (Figure 3). The predominant factor that impacts EMR data quality is the lack of cross-vendor standards (Supplementary Table, Quote A17). To mitigate this, DVs attempt to ensure records are accurately linked to a unique patient identifier (Supplementary Table, Quote A18). For claims, key quality issues are data potentially being associated with multiple conditions (Supplementary Table, Quote A19) missing information (Supplementary Table, Quote A20), or not aligning to EMR information (Supplementary Table, Quote A21). Quality mitigation for claims data involves collaboration with the data providers to research problematic claims, as well as comparisons to other data sets to help determine how to accurately code the claim for use.

#### New initiatives

New quality initiatives that DVs expect to deploy in the next 2–3 years include: (1) more technology usage including artificial intelligence (AI) (Supplementary Table, Quotes A22, A23, A24, and A25) to improve quality assessment processes, procurement, intake, and integration with current data management solutions (Supplementary Table, Quote A26); (2) further use of NLP (Supplementary Table, Quote A27); (3) patient matching improvement (Supplementary Table, Quote A28); and (4) more use of electronic patient reported outcomes (ePROs) (Supplementary Table, Quote A29).

### Addressing interoperability

#### Interoperability definitions

When subjectively defining interoperability, participants’ opinions relate to: (1) integration of processes or systems used to connect data (Supplementary Table, Quote B1), (2) the integration of multiple and disparate data sets (Supplementary Table, Quote B2), or (3) the standard by which data is integrated or delivered to customers for integration with the customer’s own data (Supplementary Table, Quote B3).

#### Interoperability standards, technologies, and their usage

Sixty-seven percent of participants use interoperability standards or technologies at their organization that allow their data to be linked to other data sources. Over 50% leverage Health Language 7 (HL7^®^) Fast Healthcare Interoperability Resources (FHIR),^29^ with one-third using Sustainable Medical Applications and Reusable Technologies (SMART)^30^ on FHIR. In terms of processes, one-third of participants use a vendor to provide data links to licensed data. Half of the participants stated their organization does not link data assets using interoperability technologies prior to licensing to customers. Six (or 50%) use interoperability technologies prior to licensing. Of these, 80% stated that data from each source are always linked as part of their organization’s standard business model (Supplementary Table, Quote B4). D/ALD can be linked to other data sources using unique tokens and keys, as well as via online portals. Security and privacy policies are important considerations when linking data sources. DVs use third-party vendors to de-identify/anonymize data and protect the token that identifies the data source to mitigate the risk of patient identification. Some DVs work with government, academic, and technology agencies to help define interoperability standards in the marketplace.

## DISCUSSION

Data quality is important for life sciences research, and standards are poorly defined.^31^ Even less is known about how DVs characterize, conduct, and communicate data quality evaluation practices to those that purchase D/ALD. Digital healthcare data sources continue to evolve in diversity and grow in volume and require greater agility to manage. For example, use of digital/virtual and remote tools has expanded during COVID-19 given the growth in use of remote clinical trials.^32^ These tools support improved direct capture of digital information for further use in RWD studies. Stakeholders who both sell and use data must put in place the right skills and expertise to ensure that data quality is assessed.

As more healthcare data technology, automation, AI, and analytics innovations emerge, it is important to understand data quality assessment practices from those who procure, curate, sell, and use D/ALD used for research purposes. Given the potential uses of digital healthcare data for RWE,^7^ RWD data quality evaluation practices must be considered when results of RWE are used for decision-making.

Our study provides new insights into how DVs recognize that D/ALD is inherently complicated. Compared to structured definitions and harmonized terminologies used for data quality assessment in academic communities,^10^ we found that DVs have standard-less, high-level definitions of data quality assessment across the data life-cycle.^33^ Only a few DVs in our study used terms such as completeness, missingness (sic), accuracy, or conformity when defining their data quality assessment processes. Most DVs describe their orientation to data quality evaluation as a direct result of the highly complex nature of the data sources and the necessary complexity of the techniques, processes, and approaches used to construct data sets required by their customers. The focus on, and rigor in, data quality assessment, particularly for RWD used to create RWE in research and regulatory decision-making, will require more structure and standards as the use of D/ALD is expected to expand.

Interoperability remains a challenge in healthcare information technology,^34^ yet growing efforts in standards setting and applications for a range of use cases hold promise. Various standards and technologies such as HL7^®^ FHIR, SMART on FHIR, or government body laws and initiatives^35^^,^^36^ continue to be created for better data access, use, and sharing/exchange in the marketplace. Our study provides insights into how DVs apply interoperability for D/ALD, and the potential seen in these evolving technologies for unique business models. How DVs address interoperability, including efforts made to improve D/ALD, will be further scrutinized and evaluated as interoperability continues to be a factor in how data are linked and shared for healthcare research, patient care, public health, decision-making, and business objectives.

Our findings revealed that DVs are more than just transactional vendors of D/ALD for research and other purposes. They are active participants in research that, like licensees, operate in a context with poorly defined standards for data quality assessments. DVs are integral to the RWE research ecosystem and must collaborate to define and practice data quality assessment standards and processes with life sciences partners.

### Limitations

This study was a discovery investigation, in a subject with limited peer-reviewed background, to understand a broad concept and term: “data quality” from the perspective of DVs. We did not concurrently study consumers of D/ALD, ie, life sciences/pharma researchers, because the goal of the study was not to perform a comparative study of data quality definitions. Definitions for data quality and data quality assessment approaches continue to evolve and do not have standards. We used as a guide, definitions of harmonized data quality assessment terms developed by academicians,^37^ and did not assume that these terms were universal, particularly among DVs. We assumed that data quality assessments by life sciences companies exist, however wanted to understand DVs perspectives of the degree to which data quality assessment practices are aspirational or commonplace (or both), and discover what techniques and resources are in place (or planned) to perform them. The variation in responses from our participants suggests that, although business and profit motives could drive responses to questions, DVs define and assess data quality in a range of ways, however, not necessarily linked to either industry or academic standards.

In this type of emerging and highly specific market of vendors supplying D/ALD, the research universe itself is limited, versus a larger universe of entities and companies who would purchase D/ALD. Despite the sample size, our study provides insights from a third of the emerging database vendor industry. Expanded research in the future could be conducted with buyers in terms of their views and experiences interacting with DVs, with greater volume of qualitative interviews or potentially a quantitative survey.

### Recommendations

Our study provides new insights into how the RWE generation research community, which includes the fast growing and minimally regulated data vendor industry, can benefit from further collaboration on data standardization for more effective and efficient data quality assessment. All stakeholders across the data life cycle (from data generation to reuse), eg, EMR companies, DVs, users (eg, pharmaceuticals, payers, providers), and regulators must better define and determine data element standards so that quality standards for D/ALD can improve collaboration between sellers of D/ALD and those who must conduct high-quality research and generate RWE. Stakeholder partnerships could relate to applying existing data quality frameworks to co-create/develop data quality assessment processes based on fit for purpose of RWE research questions,^38^ as well as data documentation guidelines, standards, and ontologies.^39^ Greater use and development of open data standards could improve data integration and interoperability of various RWD sources. The current lack of consistently applied interoperability standards in the market does not appear concerning for DVs but perhaps should be for researchers. It may be prudent for life science/pharma researchers, and other users of D/ALD, to formally include the question of the use and application of interoperability standards in their conversations and collaborations with DVs when purchasing D/ALD. DVs can also communicate their interoperability strategies more clearly to life sciences/pharma.

For companies and organizations that license and use D/ALD as part of RWD/RWE research, viewing DVs as a collaborator can directly support successful outcomes of these undertakings. D/ALD clients should seek to build a relationship and have an openness to work together and leverage vendor internal scientific teams. There does appear to be an active effort on the part of DVs to proactively collaborate to improve the customer’s final product or desired outcome.

## FUNDING

This project was funded by Merck Sharp & Dohme LLC, a subsidiary of Merck & Co., Inc., Rahway, NJ, USA.

## AUTHOR CONTRIBUTION

CEJ and DC developed the study design. All authors created the data gathering tools. DC and SV collected the data. All authors contributed to data analysis and interpretation. All authors assisted in drafting the work. CEJ, DAR, and DC revised the work critically for important intellectual content. All authors have approved its submission and agree to be accountable for all aspects of the work in ensuring that questions related to the accuracy and integrity of any part of the work are appropriately investigated and resolved.

## SUPPLEMENTARY MATERIAL

Supplementary material is available at *JAMIA Open* online.

## Supplementary Material

ooac093_Supplementary_Data

## Data Availability

The data underlying this article cannot be shared publicly to ensure the privacy of individuals and organizations that participated in the study. The data will be shared on reasonable request to the corresponding author.
