# Supplementary material for: De-identified data quality assessment approaches by data vendors who license data to healthcare and life sciences researchers
Source: JAMIA Open. 2022 Nov 2;5(4):ooac093. doi: 10.1093/jamiaopen/ooac093 (PMC9629893; doi:10.1093/jamiaopen/ooac093)
Supplement: ooac093_Supplementary_Data [file ooac093_supplementary_data.docx]

**Supplementary Table** Supportive Quotes by Objective Concept

| **Quote Code** | **Supporting Participant Quotation** | **DV ID** | **Position** | **Area of Work** |
| --- | --- | --- | --- | --- |
| A1 | "Data quality has got different dimensions so far as to how complete the data is and how recent the data is – if the data is from years ago, the data might be limited to allow the researcher to understand recent developments; how representative the data is and if it is a biased sample or the results of another data that is used so that data can be generalized for the broader population. The clinical depth of the data; does it have specific variables such as endpoints or important prognostic factors that really allow the researcher to understand the patient cases. These are different dimensions of quality that I can think of." | 6 | Director | Data Management |
| A2 | "We define data quality in large part based on its variances – variances between the data that's produced versus the data that's intended to be produced; variances between the process used to produce the data and the process by which the data should have been produced; variances between the actual use of data and the intended use of data; and then also variances between the interpretation of data and the intended interpretation of data." | 9 | Director | Engineering |
| A3 | "We have several different markers for data integrity that make use primarily of the FDA ALCOA+ data integrity markers." | 9 | Director | Engineering |
| A4 | “There is going to be some variability based on the specific application and use case that we have. When we do our assessment to see what we are looking for, we perform the analytics test to understand the data applications. We look for the cleanliness of the data or how standardized the data is, which again depends on the source of the data.” | 3 | Executive management such as vice president | Data Sciences |
| A5 | "We are extremely transparent with them and we collaborate with them on understanding the quality and it's a partnership. We have clients who are really smart and on top of their game, and they're trying to help patients and it's very important to be transparent with them and we work together with them on understanding the quality and how we can do better." | 6 | Director | Data Management |
| A6 | "We don't get into the specifics of our pattern, specific methodology is around our patient anonymization, but we do share at a high level all of that type of information about the general steps that will take place. We might not share the specific variables that are going into a methodology because we see that as IP, but, I mean, we will explain the process and the steps." | 3 | Executive management such as vice president | Data Sciences |
| A7 | "Improving interactions is always key. Being in a virtual world now, virtual meetings [should occur as much as possible] to facilitate really clear and good communication. Having...really clear expectations on exactly what data is needed and why the data is needed [is important]. There is a lot of data out there coming in [and] being collected, but really understanding...how it's going to be applied or why it's going to be needed can help...ensure that they're getting exactly what they need." | 10 | Director | Research |
| A8 | "Missingness (sic). So, we get a lot of clients going like, "Well, what is the missingness level of your data?" And that is probably the trickiest question because if there's something that's not there in a patient's record, is it truly because we're missing the data? Or is it because the patient didn't go to the hospital? Was it because they were truly healthy and didn't have an outcome? They truly didn't have an event? They were fine and they were healthy? It's one of those things that's difficult to prove. What we do is we take the data as a whole and do a lot of representative exercises with it from small scale to large scale, but we do try to replicate similar findings that are recent so we can take other real-world evidence studies and show our patient count." | 4 | Executive management such as vice president | Research |
| A9 | "That would be usually the governance of the data – how it’s maintained, how is the privacy of patients being protected , making sure that the data cannot leak in the case of multitenant solutions from one tenant or client of ours to another client, which would be disastrous." | 9 | Director | Engineering |
| A10 | “…they’re trying to validate what our process is with regards to our software: ‘What does our software do? How was our software written? What criteria our software capture[s]?’ Which I can’t get into on the phone with you [the interviewer], unfortunately, since its proprietary; but, with the partners we do define that for them and exactly how we use that proprietary software to check the quality of the data and to verify and validate the quality throughout again.” | 11 | Director | Data Sciences |
| A11 | "A common issue which is more than natural issue with real-world data is that patients may go from one side of care to another, switch from one insurance plan to another and may move from one part of the country to another. And so, as a person essentially lives their life in variety of scenarios and circumstances that can occur, how can a company provide an ALD or real-world data, then reflect what’s actually happening?" | 5 | Director | Data Sciences |
| A12 | “When it comes to metrics, the first would be data consistency. Second thing is conformity to know if the data conforming to what we are looking for. Third thing is timeliness. Fourth thing would be precision. Fifth thing is relevance. Last but not the least, the sixth thing is uniqueness.” | 1 | Director | Data Sciences |
| A13 | "Well, we're using a lot more machine learning and AI on the front end to consume and evaluate the data as a way of speeding up the evaluation, so that we're not doing it manually, although we have as I mentioned, standard methodologies and written programs to do that, it still requires a fair amount of manual intervention by a data science team." | 3 | Executive management such as vice president | Data Sciences |
| A14 | "We go through a number of processes. The quality is defined first by the fields that are entered into our data. So, we have both data science and medical science staff that define the fields that will go into the data because obviously, the better the quality of the data going into the fields, the better the outcome.” | 11 | Director | Data Sciences |
| A15 | “It can take months to onboard a healthcare organization and bring them into our data network just for all that validation purposes and some of the work that needs to be done on the healthcare organizations to get their data up to speed and in line with our quality assessment.” | 4 | Executive management such as vice president | Research |
| A16 | “I guess we trust Epic or Cerner … as best as anyone can really. It’s as good as the data that the physicians or providers are actually entering in.” | 2 | Director | Data Management |
| A17 | "EMR is the hardest because there’s no standard, just sort of standard formatting for EMR. … So, we do a lot of work upfront starting with just educating and really discussing very transparently what the data is and what some of the pitfalls may be, then we just must spend a lot of time, and again this is where some of the machine learning and AI is starting to come in." | 3 | Executive management such as vice president | Data Sciences |
| A18 | "Ensure that the electronic medical records are accurately linked to the patient and consolidated all the EMR based off of the patient ID. That's very important because the EMR data is very messy. You need to make sure that it's linked to the patient in order for it to be actionable. This is a big QC step that can be taken towards EMR to enhance its use case because EMR itself isn't helpful. It's only helpful when it's linked to a patient." | 12 | Director | Data Management |
| A19 | "I think claims data is actually difficult to validate. Because the claim can be for multiple different reasons and there can be associated conditions. So, it is very difficult to pin down and say, 'this claim is for this.' Sometimes you can’t tell that…this is where the digital asset management comes into play. On an average, like 70% of the time, this is actually prescribed per level availed for the whole kidney condition and only 30% of the time it is prescribed to care for the whole blood pressure. So, we use that parameter to make our decision." | 1 | Director | Data Sciences |
| A20 | "I would say Claims is probably the most difficult. I think everything else is pretty much the same, but claims data is far more difficult because I think there's a lot of missing data there and that's why the claims actually didn't go through." | 2 | Director | Data Management |
| A21 | "I would say the claims data is more difficult to validate because of the manner in which it is entered and then transferred to us. The EMRs are much more aligned to our system because the system was built originally for that purpose. Claims are something we added in the last year and a half that have completely different structures in their data and as I said earlier, they don't always align to what is in the EMR even though you would suspect it should. They are different fields that we often have to deal with in claims data particularly related to prior drug therapies, coding and such things that are in an EMR that we have to pour out and separate off and put kind of into their own data set, and then pull that clinical data forward and match it to the EMR wherever we can. If that's not available, then the claims represent the patient as a stand-alone data set." | 11 | Director | Data Sciences |
| A22 | "One of the many initiatives is machine learning and AI. A lot of manufacturer pharmaceutical companies and life science companies are trying to leverage more AI-driven solutions to do a lot of the groundwork. So, one QC can be implemented by using a strong querying software to check the data." | 12 | Director | Data Management |
| A23 | "Over the next two years, we'll be moving from data information assets that are analytic products of using that data, applying data science and data analytics. I've already briefed the CEO, the Chief Data Officer here as well as my boss – the Chief Information Officer – on various business cases that we'll make use of things like artificial intelligence, machine learning, as well as data science and analytics." | 9 | Director | Engineering |
| A24 | "I would say over the next two to three years, it's the implementation of more technology to validate data accuracy and data consistency, as well as because technology will take a bigger role in the actual curation of data." | 5 | Director | Data Sciences |
| A25 | "I think for us as an organization, we are moving to use more single points of entry to ensure that data quality, and using artificial intelligence and/or bots for moving data where it needs to go ultimately in the end, so then that way, there's no, there's not a lot of changes or room for errors in between." | 10 | Director | Research |
| A26 | "AI and machine learning are really where we're focusing a lot of our efforts around data quality and data procurement as well as loading the data and integrating it into our warehouses and data lake." | 3 | Executive management such as vice president | Data Sciences |
| A27 | "So, I would imagine that in the next two, three, five years, we will be seeing an increase in the use of NLP to extract information from the patient’s charts, [thereby] increasing the completeness of the data." | 6 | Director | Data Management |
| A28 | "We need to make sure that there are just a lot more patients who have richer data and that we're pooling all the data that's available in the EMR and working with our healthcare organizations to make sure that is provided in that data warehousing. We are working on integrating [a] clean history of closed claims network without EMR data. We have this already in the works, it's just we haven't really commercially released it yet. This linked data will be a great high quality data network [as] we are matching the same patients who have EMR data with their claims data, and it's really helping with the missingness that people are always concerned about because we can use the pharmacy data that's from the claims to fill in the EMR data that we may not have." | 12 | Director | Data Management |
| A29 | "And also, we might see more PROs – Patient Reported Outcomes. Everyone has a cell phone nowadays and they use smart watches; we can use more self-reported outcomes. How is the patient? How the patient is feeling, etcetera. So, I think companies will start to leverage this type of data going forward." | 6 | Director | Data Management |
| B1 | "There are multiple different processes that we have. So, it is essentially the integration between the processes to really come up with a consistent answer. That’s how I actually define it as interoperability." | 1 | Director | Data Sciences |
| B2 | "Interoperability kind of applies across our whole organization because we’re constantly connecting to disparate sources, both inbound and outbound. We’re always making sure that the [data is] always maintained. We [are] effectively the hub for all of that data. Our operations team are the ones that maintain that process for us,...through everything I’ve already mentioned in longitudinally across projects once they are started. That [is] how we would define it." | 11 | Director | Data Sciences |
| B3 | "In my opinion, it is all about linking the data so that it can be operationalized and sold to our customers." | 12 | Director | Data Management |
| B4 | "When we sell them to pharma, they’re always linked because that is part of our business model. We always link all the various data sets we have for different subtypes, in most cases cancer subtypes with molecular data and the therapeutic data." | 11 | Director | Data Sciences |
